# A Randomized Controlled Clinical Trial Comparing the Use of High Purity Type-I Collagen-Based Skin Substitute vs. Dehydrated Human Amnion/Chorion Membrane in the Treatment of Diabetic Foot Ulcers

**DOI:** 10.7759/cureus.75182

**Published:** 2024-12-05

**Authors:** Naveen Narayan, Suhas Gowda, Chethan Shivannaiah

**Affiliations:** 1 Plastic Reconstructive and Aesthetic Surgery, Adichunchanagiri Institute of Medical Sciences, B. G. Nagara, IND

**Keywords:** advanced wound care, dehydrated human amnion/chorion membrane allograft, dermal substitute, diabetic foot ulcers (dfus), helicoll®, high purity type-1 collagen-based skin substitute (hptc), type 1 collagen

## Abstract

Diabetic foot ulcers (DFUs) are a prevalent and costly complication of diabetes, contributing significantly to patient morbidity and healthcare burdens. This randomized controlled trial aimed to compare the safety and efficacy of high-purity type-I collagen-based skin substitute (HPTC) and dehydrated human amnion/chorion membrane (dHACM) in the treatment of DFUs. The study enrolled patients from the Adichunchanagiri Institute of Medical Sciences (B.G. Nagara, KA, IND) and followed them for a four-week treatment period, with wound healing outcomes evaluated on days seven, 10, 14, 17, 21, and 28. A total of 28 patients were randomized to receive standard care with either HPTC or dHACM.

The results demonstrated that the HPTC group achieved significantly better healing outcomes, with 85.71% of patients exhibiting ≥50% wound size reduction at four weeks compared to 50% of patients in the dHACM group. Furthermore, complete wound closure was observed in 10 patients treated with HPTC compared to seven in the dHACM group. The mean reduction in wound size was 86.48% in the HPTC group, compared to 77.70% in the dHACM group. The superior healing effect of HPTC is attributed to its composition, which enhances cellular attachment and accelerates tissue regeneration. In conclusion, HPTC demonstrated faster and more complete healing of DFUs compared to dHACM, indicating its potential as a more effective treatment option for managing chronic DFUs and reducing the risk of long-term complications.

## Introduction

Chronic wounds, particularly diabetic foot ulcers (DFUs), impose a substantial financial burden on healthcare systems. The direct costs of treating chronic wounds in the United States are estimated to be around $30 billion annually. Diabetic foot ulcers are the most common type of lower extremity wound, accounting for approximately 80% to 85% of these cases. Annually, between 6.5 and 7 million individuals in the United States are affected by chronic DFUs. With an aging population and increasing incidence of risk factors such as obesity and congestive heart failure, the prevalence of DFUs is expected to rise [1].

In a recent meta-analysis (2024) of 18 studies involving a total of 55,988 people with diabetes, the pooled prevalence of DFUs in India was estimated at 6.2% (95% CI: 4.0; 9.4%). Regional analysis indicated similar prevalence in the regions to the East (9.5%), South (7.4%), and North (5.6%) of India (p = 0.42). Hospital-based studies exhibited a higher prevalence (7.5%) compared to community-based studies (2.5%) (p = 0.02) [2]. Out of 62 million diabetics in India, 25% develop DFUs, of which 50% become infected, requiring hospitalization, while 20% need amputation. Diabetic foot ulcers contribute to approximately 80% of all non-traumatic amputations in India annually. Patients with a history of DFU have a 40% higher 10-year death rate than those without [3].

Chronic DFUs are associated with significant morbidity and reduced quality of life, as the healing process is typically prolonged and painful. Even under the best of circumstances, these ulcers may take weeks or months to heal, often following a frustrating cycle of slow healing and recurrent breakdown. Wound care specialists frequently encounter patients who have endured these ulcers for years, with some facing amputation as their only option for pain relief [4].

Diabetic foot ulcers are notoriously hard to heal. However, when treatment is provided along the evidence-based practice guidelines wherein there is recognition and management of infection and ischemia, most patients are observed to achieve healing. The above should be complemented with wound debridement, pressure offloading, and suitable patient education [5].

Several mechanisms are involved in the development of DFUs. These are neuropathy, increased biomechanical stress, external trauma, and peripheral arterial disease (PAD), along with immune suppression [6]. Also, DFUs are often complicated by infection [7]. Thus, DFUs have different clinical presentations, and hence, management strategies and outcomes are influenced by factors such as PAD, infection, and, probably, comorbidity [8]. Diabetic patients, though, with ischemic foot ulcers not available for revascularization are not completely excluded from healing without major amputation [9]. Also, the presence of DFUs is strongly associated with an increased risk of death in diabetics [10].

The amnion and chorion membranes of the human placenta have long been used in the clinical setting in the treatment of DFUs. Their observed efficacy is owed to their natural role as a barrier with self-restorative properties [11]. Type-I collagen, when it is a full molecule, purified, and uncross-linked, provides 3,000 receptor sites per molecule for growth factors such as fibroblasts to attach to, making it an excellent matrix for wound healing. Type-I collagen is 97% similar across different species, whereas type-II and type-III collagen are only 80% similar within the same species. For example, type-I collagen in humans is very similar to that in cows or birds. Type-I collagen is the least immunogenic, as it does not provoke an immune response due to its lack of the sulfur-containing amino acid cysteine. In contrast, type-II and type-III collagen contain higher concentrations of cysteine, making them more immunogenic [12].

The purpose of the present study is to compare the safety and efficacy of high-purity type-I collagen-based skin substitutes (HPTC), which are free from contaminants such as lipids, elastin, and other immunogenic particles, versus dehydrated human amnion/chorion membrane (dHACM) in the treatment of DFUs. This randomized, controlled clinical trial aims to provide insights into the optimal treatment approach for improving healing outcomes and reducing the burden of chronic DFUs.

## Materials and methods

We conducted a randomized, controlled open-label study to compare the safety and efficacy of the HPTC Helicoll® versus dHACM in the treatment of DFUs. The study involved patients with DFUs under the care of wound care specialists at Adichunchanagiri Institute of Medical Sciences (B. G. Nagara, KA, IND). The study was overseen by the primary investigator, author Narayan N. The study received approval from the Institutional Ethics Committee of Adichunchanagiri Institute of Medical Sciences (approval no. EC/NNEW/INST/2023/KA/0382) and was preregistered on ClinicalTrials.gov (identifier: NCT06470087; protocol record: HPTC101). Informed consent was obtained from patients with a signed consent form before study-related procedures were conducted. The investigator adhered to the applicable regulatory requirements and good clinical practice in obtaining and documenting informed consent. All study products were handled, and stored in compliance with good practices. Patient confidentiality was rigorously maintained. Patients were randomized using a simple lottery method.

Patient screening and eligibility

The study population consisted of patients seeking treatment for DFUs. Eligible patients were those willing to participate and comply with scheduled visits on days 7, 10, 14, 17, 21, and 28. The study included two phases: screening and treatment. The screening phase aimed to determine patient eligibility for the treatment phase. The inclusion and exclusion criteria are detailed in Table 1. During the screening, a series of assessments were conducted, including demographics, medical history, concomitant medications, vital signs, physical examination, leg ulcer history, clinical infection signs at the ulcer site, and ankle-brachial index measurement.

**Table 1:** Inclusion and exclusion criteria DFU: Diabetic foot ulcer

Inclusion criteria	Exclusion criteria
Subjects must be at least 18 years of age or older.	A subject known to have a life expectancy of less than six months.
Subjects must have a diagnosis of type 1 or 2 diabetes mellitus.	If the target ulcer is infected or if there is cellulitis in the surrounding skin.
At enrolment, subjects must have a target DFU with a minimum surface area of 5.0 cm² and a maximum surface area of 10.0 cm², measured post-debridement using a ruler.	Presence of osteomyelitis or exposed bone, probes to bone or joint capsule on investigator’s exam, or radiographic evidence.
The target ulcer must have been present for a minimum of four weeks and a maximum of 52 weeks of standard care prior to the initial screening visit.	A subject that has an infection in the target ulcer that requires systemic antibiotic therapy.
The target ulcer must be located on the foot, with at least 50% of the ulcer below the malleolus.	A subject receiving immunosuppressants (including systemic corticosteroids at doses greater than 10 mg of prednisone per day or equivalent) or cytotoxic chemotherapy.
The target ulcer must be full thickness on the foot or ankle and must not probe to the bone.	Topical application of steroids to the ulcer surface within one month of initial screening.
Adequate circulation to the affected foot as documented by any of the following methods performed within three months of the first screening visit: a. Transcutaneous oxygen measurement (TCOM) ≥ 30 mmHg; b. Ankle-brachial index (ABI) between 0.7 and 1.3; c. Peripheral vascular resistance (PVR): Biphasic; d. Toe-brachial index (TBI) ˃ 0.6 e. Alternatively, arterial Doppler ultrasound can be performed to evaluate biphasic dorsalis pedis and posterior tibial vessels at the level of the ankle of the target extremity.	A subject with a previous partial amputation on the affected foot is excluded if the resulting deformity impedes proper offloading of the target ulcer.
If the subject has two or more ulcers, they must be separated by at least 2 cm. The largest ulcer satisfying the inclusion and exclusion criteria will be designated as the target ulcer.	A subject with glycated hemoglobin (HbA1c) greater than or equal to 13%, measured at or within three months of the initial screening visit.
The subject must consent to use the prescribed off-loading method, i.e., offloading slab using plaster of Paris.	A subject with a serum creatinine ≥ 3.0 mg/dL within six months of the initial screening visit.
The subject must agree to attend the twice-weekly/weekly study visits required by the protocol.	A subject with an acute Charcot foot, or an inactive Charcot foot that impedes proper offloading of the target ulcer.
The subject must be willing and able to participate in the informed consent process.	Women who are pregnant or considering becoming pregnant within the next six months.
Patients must have read and signed the informed consent form (ICF) before screening procedures are undertaken.	A subject with end-stage renal disease requiring dialysis.
	A subject who participated in a clinical trial involving treatment with an investigational product within the previous 30 days.
	A subject who, in the opinion of the investigator, has a medical or psychological condition that may interfere with study assessments.
	A subject treated with hyperbaric oxygen therapy or a cellular and/or tissue product (CTP) in the 30 days prior to the initial screening visit.

Study treatment

The treatment phase of the study began with a series of assessments to confirm patients' continued eligibility. Subjects who met the study's inclusion criteria after the screening period were randomized into one of two groups: (1) application of standard of care (SOC) with HPTC; and (2) application of SOC with dHACM. In this study, neither the patients nor the clinicians were blinded to group assignments. The randomization schedule was balanced and permuted in blocks of 12. When a patient was ready for randomization, the study site contacted a representative from the sponsor, who then opened a sequentially numbered opaque envelope to reveal the group assignment, ensuring allocation concealment.

During the four-week treatment phase, patients were re-evaluated on days 7, 10, 14, 17, 21, and 28. The SOC bandage included a three-layer dressing system. The first layer had a non-adherent and porous paraffin gauze, the second layer had absorbent gauze pads, and the third layer involved a soft roll and crepe bandage. The following sizes of HPTC and dHACM are easily available commercially and were used in our study: 0.8 in x 1.6 in or 2 cm x 4 cm (8 sq cm) and 1.6 in x 1.6 in or 4 cm x 4 cm (16 sq cm) HAPTC; and 5 cm x 5 cm (25 sq cm) and 8 cm x 6 cm (48 sq cm) dHACM.

If the study ulcer was found to be 100% re-epithelialized during the visit, no further study procedures were conducted at that time. The patient was then scheduled for a follow-up visit after one week to confirm the healing. If complete healing was not observed, an assessment was performed to check for signs of clinical infection. If an infection was diagnosed, treatment with topical antimicrobials (betadine, chlorhexidine) or oral antibiotics was allowed, but the use of topical antibiotics (erythromycin, polymyxin, mupirocin) was prohibited.

Following the infection assessment, the ulcer was cleaned, photographed, and debrided at the investigator's discretion to ensure a clean, granulating ulcer base with minimal adherent slough. The SOC was then reapplied, and the patient was instructed to keep the bandaging dry. The patient was also advised to contact or visit the study site if the bandage became soiled or was removed.

Study completion

Patients completed the study four weeks after their first treatment visit. However, if a patient's study ulcer closed before the four-week mark, they were considered to have completed the study at that time. Complete healing of the study ulcer was defined as 100% re-epithelialization with no drainage. Throughout the treatment period, patients had the right to refuse participation or withdraw from the study at any time without prejudice. If a patient chose to withdraw from the study, their last recorded wound measurement was carried forward and used to calculate the change in wound size and their final outcome.

Study outcomes

Primary Endpoint 

The primary outcome of the study was the proportion of subjects who achieved improvement in wound healing, as observed on days 7, 10, 14, 17, 21, and 28. The wound closure of the target ulcer was continuously monitored until the end of the four weeks.

Secondary Endpoints

The 'time to achieve complete wound closure' is the time taken for the target ulcer to achieve complete wound closure through days 7, 10, 14, 17, 21, and 28. The 'percentage wound area reduction' was measured weekly on days 7, 10, 14, 17, 21, and 28 using digital photography. The 'mean number of repeated applications of HPTC' is the average number of times the HPTC was reapplied to achieve wound closure.

Exploratory Endpoint

The appearance, structural stability, and fragility of the newly formed skin were documented at each visit. Any recurrence of the wound was also monitored.

## Results

As shown in Table 2, the mean duration of ulcer in group A was 2.86 months, and in group B was 2.93 months. The mean ulcer sizes were 5.52 cm² and 5.21 cm² in group A and group B, respectively.

**Table 2:** Characteristics of study sample DFU: Diabetic foot ulcer

Characteristics	HPTC (Group A)	dHACM (Group B)
Mean age (years)	49.4 (42.0 - 50.0)	52.3 (44.0 - 54.0)
≥65 years (%)	0%	14.3%
Male (%)	75%	85%
Mean body mass index (BMI)	25.41 (23.9 - 27.7)	25.30 (22.1 - 27.9)
Mean DFU duration (months)	2.86 (2 - 3)	2.93 (2 - 3)
DFU duration >12 months (%)	0%	0%
Mean DFU size (cm²)	5.52 (3.0 - 7.84)	5.21 (2.8 - 7.8)
DFU Size >10 cm² (%)	14.29%	7.14%

The primary study outcome, as shown in Tables 3-4, was the proportion of patients with a ≥50% reduction of wound size at four weeks for those receiving HPTC vs. those receiving dHACM. Reduction in wound size of ≥50% occurred in significantly greater numbers of patients receiving HPTC vs. those receiving dHACM (12/14 (85.71%) vs. 7/14 (50%); p = 0.245).

**Table 3:** Group A reduction in wound size

Patient	Baseline	Day 28	Percentage of reduction
ES	4.00	1.73	56.75%
MN	3.00	0.00	100%
SK	2.50	0.00	100%
RW	7.84	1.40	82.13%
KM	10.00	0.00	100%
RJ	6.80	1.40	79.41%
VN	3.80	0.00	100%
RA	2.60	0.00	100%
ST	6.84	0.00	100%
RK	10.60	0.80	92.45%
IP	5.60	0.00	100%
MD	3.00	0.00	100%
SR	2.50	0.00	100%
MM	7.84	0.00	100%

**Table 4:** Group B reduction in wound size

Patient	Baseline	Day 28	Percentage of reduction
NA	4.80	0.00	100%
MG	2.80	0.00	100%
SA	4.20	1.00	76.19%
RZ	6.20	4.20	32.26%
KR	7.80	5.80	25.64%
VA	2.80	0.60	78.57%
ME	3.80	0.00	100%
SV	2.60	0.00	100%
KA	8.20	6.20	24.39%
PA	10.60	3.80	64.15%
VK	5.60	0.00	100%
MJ	3.00	0.40	86.67%
SU	2.50	0.00	100%
BH	7.84	0.00	100%

The calculated t-statistic, based on the total study population as specified in Table 5, shows a very significant difference in the percentage of wound reduction between the two groups. The p-value is effectively 0 indicating that HPTC (93.62 ± 0.12) leads to effective wound healing compared to dHACM (77.71 ± 0.28).

**Table 5:** The mean and SD of percentage of wound reduction in the total study population

Groups	Sample size (n)	Mean ± SD percentage of wound reduction
A	14	93.62 ± 0.12
B	14	77.71 ± 0.28

**Figure 1:**
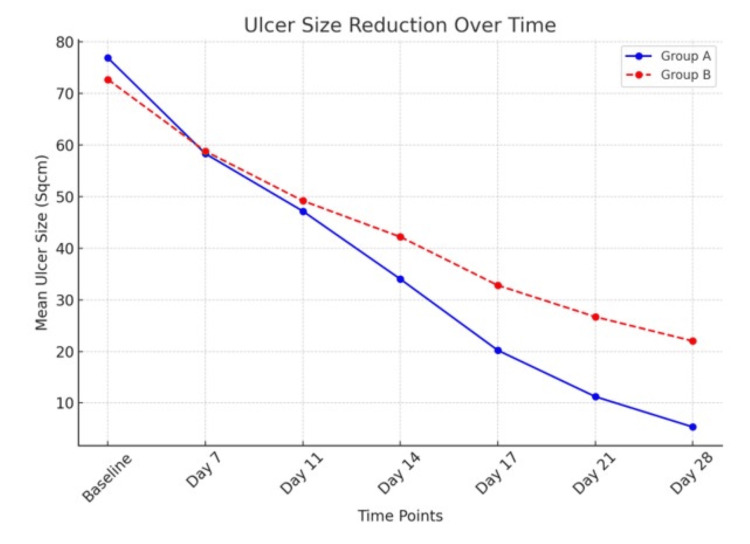
Ulcer size reduction over time

As shown in Table 6, patients receiving HPTC had a mean reduction in DFU size over the four-week study period of 86.48% compared with 77.70% in the dHACM group. The mean percentage of reduction in DFU size on days 7, 10, 14, 17, 21, and 28 are presented in Figure 1. Note the increased rates of healing in the HPTC group compared with those patients receiving dHACM during the four-week study period.

**Table 6:** The mean percentage of reduction in ulcer size

Groups	Mean ulcer size in sq cm (baseline)	Mean ulcer size day 7	Mean ulcer size day 11	Mean ulcer size day 14	Mean ulcer size day 17	Mean ulcer size day 21	Mean ulcer size day 28
A	76.92	58.34	47.19	34.02	20.2	11.22	5.33
B	72.74	58.82	49.2	42.2	32.8	26.7	22

**Figure 2:**
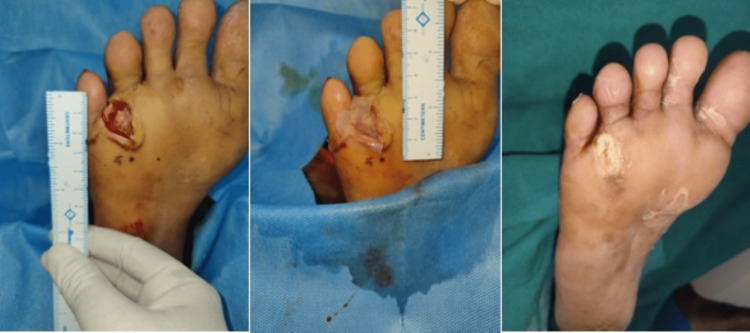
Progress of wound closure in group A with wound healing on day 17 From left to right: DFU over forefoot region; Helicoll® applied; wound healing noticed on day 17 DFU: Diabetic foot ulcer

**Figure 3:**
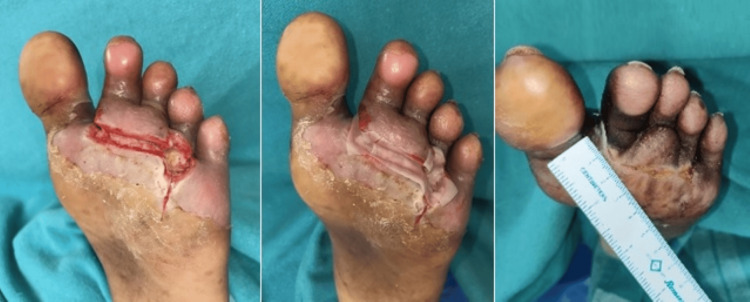
Progress of wound closure in group A with wound healing on day 21 From left to right: DFU over forefoot region; Helicoll® applied; wound healing noticed on day 21 DFU: Diabetic foot ulcer

**Figure 4:**
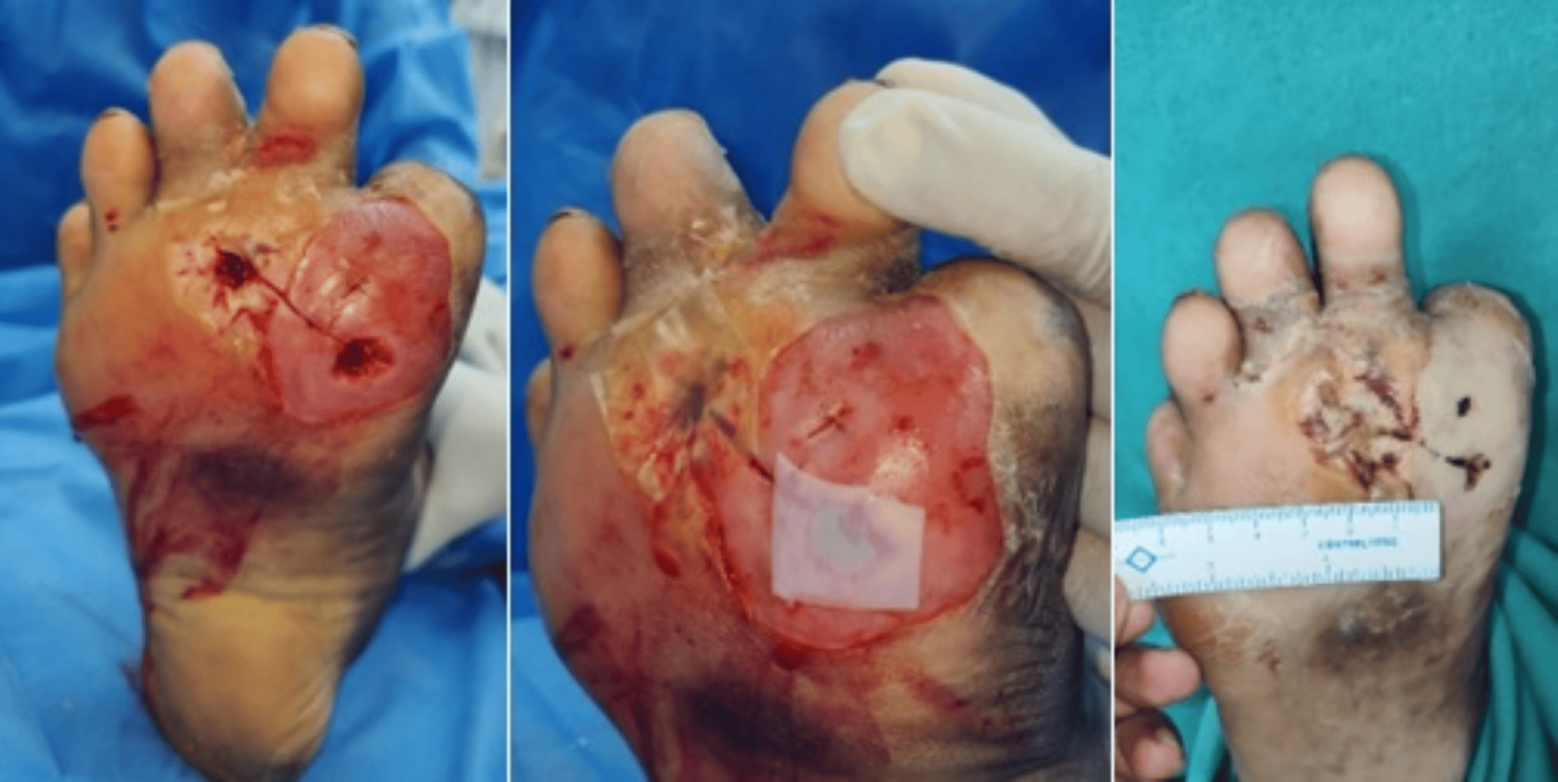
Progress of wound closure in group B From left to right: DFU over forefoot region; dHACM applied; wound status on day 28 DFU: Diabetic foot ulcer, dHACM: Dehydrated human amnion/chorion membrane

Within the HPTC group, the wound area was reduced by a mean of 5.48 ± 2.32 cm² during the study period (from randomization to the end of the study). For those patients receiving dHACM, the wound area was not reduced as much during the study period, with a mean difference of only 4.30 ± 3.21 cm² between randomization and the four-week visit. In the four-week study period, 10 patients in the HPTC group and seven patients in the dHACM group had complete wound closure. The clinical photos in Figures 2-4, show the degree of wound healing with HPTC (group A) and dHCAM (group B), and the time taken to achieve it.

## Discussion

This study is one of the first randomized trials to compare the efficacy of HPTC with dHACM in the treatment of DFUs. The results suggest that DFUs treated with HPTC demonstrated significantly better outcomes in terms of wound size reduction and healing rate compared to those treated with dHACM. The healing advantage of HPTC was especially notable when comparing the mean percentage of reduction in ulcer size over the 28-day study period.

The pathogenesis of foot ulcers is often difficult and with challenging management. Knowledge of novel techniques and newer products can allow the treating physician to achieve success in their endeavor to provide positive outcomes for these patients. Advanced therapies such as bioengineered skin substitutes (Helicoll®) and wound care tools have been shown in numerous studies to accelerate the healing process in DFUs. However, there is no panacea in all these situations [13]. Treatment technologies to accelerate the healing process with a hastened healing process maintained over a longer period of time and reduced complications such as infections are advantageous in the management of DFUs [14].

Patients in the HPTC group showed a remarkable 85.71% rate of ≥50% wound size reduction compared to 50% in the dHACM group. Moreover, complete wound closure was observed in 10 of the patients treated with HPTC, compared to seven patients in the dHACM group. These findings highlight the superior wound-healing potential of HPTC in managing DFUs within a short timeframe. Additionally, the mean reduction in DFU size over four weeks was greater in the HPTC group (86.48%) compared to the dHACM group (77.70%). Reyzelman et al., in their 86-patient multicenter trial study, have demonstrated a healing rate of 69.6% with dHACM. A tad less than our study [15].

A key factor contributing to the success of HPTC is its composition of high-purity (>97%) type-I collagen, which is highly biocompatible and retains the native properties of collagen necessary for wound healing. In contrast, human intact tissue membrane-derived products (HCT/P), such as dHACM, may lose bioactivity due to chemical cross-linking, which compromises their ability to promote efficient wound healing. The bioactivation of HPTC through a phosphorylation process enhances its ability to attract cells via signal transduction, promoting the formation of new blood capillaries within days of application. This aligns with the accelerated healing observed in the HPTC group, particularly during the initial stages of treatment.

The study also highlighted the impact of HPTC on addressing glycosylation of lysine residues in hyperglycemic patients. Glycosylation can impair collagen maturation in the wound bed, and HPTC’s ability to absorb excess glucose mitigates this negative effect, supporting better wound healing outcomes in patients with DFUs [16].

In terms of healing progression, patients treated with HPTC showed significantly faster reductions in wound size across various time points. By day 28, the average ulcer size in the HPTC group had reduced to 5.33 cm² compared to 22 cm² in the dHACM group. This faster wound closure is clinically significant, as prolonged healing in DFUs increases the risk of infection and complications. Importantly, wound closure at or before four weeks is considered a strong predictor of long-term healing, and the results here support the potential of HPTC to achieve such outcomes.

In a study by Dhanraj et al., it was demonstrated that the incorporation of new blood capillaries happened during the initial four to five days of application of Helicoll® and the same was evident in the clinical outcome of our study. The reason for such advanced bioactivity of Helicoll® may be due to the phosphorylation of high-purity type-I collagen in Helicoll® [12]. Studies by DiDomenico et al. [17] and Mohammed et al. [18] show that dHACM-applied wound healing and 50% wound contraction duration were comparable to our study. But Helicoll® applied wounds healed and contracted faster compared to dHACM.

The main goal of DFU treatment is to enhance and facilitate complete wound healing; therefore, reducing the risk of complications such as infection, amputation, and delayed wound healing [19]. Helicoll® gets incorporated into the wound and provides a good matrix for cellular and vascular replacement. The possible mechanism of action of Helicoll® is attributed to moist wound bed cell signaling, early wound healing, and neovascularization.

It may be noted that with Helicoll® the native cells climb and lay down native matrix protein and collagen. The increased dermal component offers the healing wound bed more tensile strength and pliability, and hence better functional and aesthetic results. Wound covered with Helicoll® compared to skin graft application alone results in decreased formation of contractures and better pliability. The advantage of Helicoll® in the treatment of DFUs is that it is available as a cost-effective product, reduces hospitalization, and in most cases averts the need for surgery and the possible risk of amputation. In their study, Armstrong et al. report that even when a DFU is healed, the chances of recurrence concomitantly increase with an estimated possibility of 40% within one year and 65% within five years [20].

One limitation of the study was the inability to blind caregivers to treatment assignments, which could introduce bias. However, objective measurements were employed to minimize this risk, and the randomization of patients strengthens the validity of the findings. Future studies should focus on longer follow-up periods to evaluate the long-term healing rates of HPTC, as well as determine the optimal frequency and duration of its application.

In conclusion, this study demonstrates that HPTC (Helicoll®) is an effective treatment for DFUs, offering faster and more complete healing compared to dHACM. Given the high burden of non-healing DFUs and the limitations of standard care, HPTC represents a promising advancement in wound care for diabetic patients.

## Conclusions

This study demonstrated that HPTC (Helicoll®) is a highly effective treatment for DFUs, providing superior outcomes in terms of wound size reduction and overall healing compared to dHACM. The use of Helicoll® resulted in faster wound closure, with a higher percentage of patients achieving significant reductions in wound size within four weeks. These findings are particularly important given the challenges associated with the prolonged healing of DFUs, which can lead to increased risk of infection, amputation, and reduced quality of life.

The unique composition of Helicoll®, consisting of high-purity type-I collagen, enhances its biocompatibility and promotes cellular activity essential for tissue regeneration. This study highlights the potential of Helicoll® as a valuable alternative to conventional treatments for DFUs, offering a more efficient path to wound closure and minimizing the burden of chronic ulcers. Future research should explore the long-term benefits of Helicoll® and its application in broader patient populations to solidify its role in the management of diabetic wounds.
